# Multispectral Quantum Dot Tags for Advanced Anticounterfeiting Applications

**DOI:** 10.1021/acsanm.6c00386

**Published:** 2026-04-11

**Authors:** Syeda Ramsha Ali, Yueyu Guo, Soumya Sarkar, Kees de Groot, Nema M. Abdelazim

**Affiliations:** School of Electronics and Computer Science, 7423 University of Southampton, Southampton SO17 1BJ, United Kingdom

**Keywords:** Optical PUFs, Quantum Dots, Security, Nanomaterials, Photoluminescence

## Abstract

Physical unclonable functions (PUFs) based on nanophotonic materials offer a promising route toward secure and tamper-resistant authentication. Here, we introduce a quantum dots (QDs)-driven optical fingerprinting (identifier) platform that utilizes four distinct photoluminescence (PL) emission peaks generated from two cadmium-free CIS/ZnS QDs formulations deposited side-by-side. Under multiwavelength excitation, each of them exhibits a dual-peak emission response, yielding a combined four-peak, multiexcitation spectral profile. By extracting the wavelength, full width at half-maximum, and intensity from each peak across nine excitation wavelengths, we obtain 108 independent spectral features, which are converted into a 216-bit binary fingerprint. This work incorporates a features fusion strategy that compresses multidimensional spectral descriptors into compact, discriminative digital features, enabling stable, high-entropy encoding from complex PL emission behavior. Comprehensive statistical analysis demonstrates strong uniqueness with a mean inter-Hamming distance of 0.512 ± 0.028, a wide collision margin of 99−123 differing bits, and repeatability with near-zero intra-tag variation. Bit-level randomness metrics confirm near-ideal statistical behavior after binarization. The four-peaks architecture therefore represents a significant advancement over single-peak or dual-peaks luminescent PUFs, enabling dense, high-entropy fingerprints from cadmium-free materials while remaining compatible with typical readout hardware. This work establishes a foundation for next-generation optical authentication technologies using multipeak QDs emitters.

## Graphical abstract

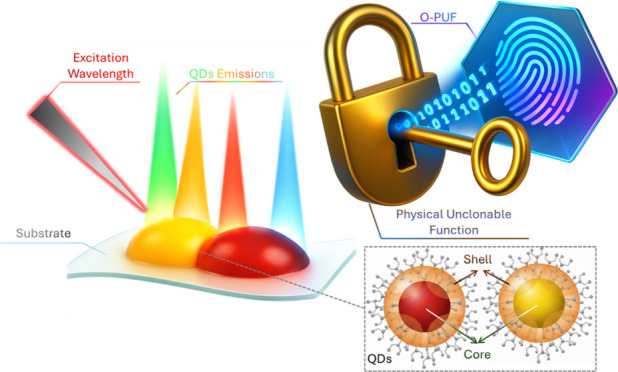

## Introduction

In recent years, the escalation of product counterfeiting across sectors ranging from consumer electronics^1^ to luxury clothing^2^ and premium arts^3^ has created profound challenges for product authentication and identity verification. The ability of adversaries to replicate packaging, clone electronic modules, and imitate branded goods with increasing precision has become a major security concern. This trend highlights an urgent need for authentication technologies that are resilient against physical reverse engineering. Conventional anticounterfeiting strategies such as barcodes,^4^ holography,^5^ and secure quick response codes^6^ have become increasingly vulnerable to duplication. These limitations have motivated the development of authentication technologies that exploit intrinsic physical randomness to generate optical fingerprints that are difficult to reproduce through the fabrication process. Physical unclonable functions (PUFs) have emerged as promising candidates, as they use intrinsic material disorder to generate irreversible responses that cannot be duplicated.^7–9^ PUF systems use stochastic variations in materials or structures to generate unique responses when stimulated by external inputs. When a stimulus (challenge) interacts with the inherent physical disorder of a system, a measurable output (response) is produced, forming a challenge-response pair (CRP) that can be used for authentication. The ability to generate many unpredictable CRPs is important for secure identification because it increases the encoding capacity of the system and reduces the probability of counterfeiting/cloning.

Among the various PUF technologies, optical PUFs represent a particularly attractive class originating from the physical one-way function proposed by Pappu et al. in 2002.^10^ Optical PUFs harness the optical material characteristics such as photoluminescence (PL) emissions to produce rich and unclonable responses.^11–13^ Their ability to generate a large number of CRPs significantly expands the encoding capacity of the system and makes it hard to clone. Additionally, as they rely on the optical response of the material rather than on electronic circuitry, optical PUFs enable noncontact interrogation and support remote authentication environments. A wide variety of materials and platforms have been investigated for optical PUFs, including bulk polymers,^14,15^ silicon photonic structures,^16–18^ plasmonic architectures,^19,20^ lanthanide-based materials,^21–23^ transition metal dichalcogenides,^24,25^ perovskite thin films,^26,27^ and quantum dots (QDs).^27–34^ Persistent phosphors embedded in flexible matrices have been used to produce mechanically strong anticounterfeiting films with ultraviolet-stimulated emission responses.^35^ Similarly, chiral photonic crystal structures can generate tunable structural colors through optical rotation effects, enabling wavelength-selective optical signatures.^13,36^ Other approaches have employed stochastic distributions of dye-doped fibers or droplets to create spatially random optical patterns that function as PUFs.^37^ While these platforms provide effective routes for generating physically unique optical signatures, they typically rely primarily on spatial randomness or structural color variations as the main source of entropy.

Semiconductor QDs offer a compelling alternative for PUFs technologies because of their solution processability, tunable bandgap, and intrinsic ability to generate diverse optical signatures.^38–40^ Their quantum-confinement nature introduces intrinsic nanoscale randomness, which naturally produces subtle differences in their optical responses. QDs offer additional advantages for spectral encoding applications, as they exhibit size- and composition-dependent PL with high brightness and tunable emission wavelengths, enabling the generation of multiple spectrally distinct peaks from nanoscale emitters. Additionally, QDs exhibit excitation-dependent behavior,^41,42^ including changes in emission wavelength and intensity, enabling multiple independent optical features to be extracted for producing unclonable optical fingerprints (identifiers). Generating binary codes is central to practical authentication systems, as the security of optical PUF tags depends on their ability to transform physical randomness into a large and unpredictable digital strings. This capability is particularly important in systems that require extensive use of CRPs. Recent efforts have demonstrated QDs-based optical PUFs employing color multiplexing and stochastic photonic combined structures to enhance the number of CRPs, but most of them rely on using toxic heavy-metal QDs, such as cadmium (Cd)-based QDs.^33,43^ These systems raise concerns regarding toxicity and suitability for consumer-related applications. These challenges have motivated a shift toward environmental friendly alternatives such as copper indium sulfide/zinc sulfide (CIS/ZnS) QDs.^44,45^ CIS/ZnS QDs are composed of low-toxicity elements, making them suitable for use as security inks in consumer products without posing health or environmental risks. Beyond their low toxicity, CIS/ZnS QDs offer additional properties that make them well suited to optical PUFs. They can achieve high PL quantum yields through effective surface passivation, and their emission can be tuned across the visible to near-infrared spectrum via composition and size control.^46,47^ The defect-rich, off-stoichiometric nature of CIS also introduces multiple recombination pathways, producing intrinsic optical variability that can support high encoding capacity for fingerprint generation.^48–50^ This property is advantageous for spectral multiplexing and high-dimensional encoding of optical PUFs. Their compatibility with scalable synthesis and deposition techniques further enhances their commercial viability.

In this work, we introduce a simple deposition strategy of side-by-side droplets to enhance the encoding capacity of QDs-based optical fingerprints using two spectrally distinct colored formulations of CIS/ZnS QDs. Instead of chemically mixing them, we deposit each drop side-by-side, allowing their independent PL emissions to be recorded simultaneously under certain excitation conditions. This lateral deposition offers a composite four-peak PL emission spectrum, effectively doubling the available spectral features without altering the material chemistry. The resulting multimodal emission is processed using computational algorithms to extract peak-dependent parameters, which are then converted into high-entropy optical fingerprints. This strategy provides a simple yet powerful route to expand the encoding capacity of QDs-based PUFs, enabling complex unclonable spectral signatures for secure authentication.

## Results and Discussion

The schematic in Figure 1a illustrates the preparation strategy and the underlying motivation of the lateral QDs side-by-side deposition approach. Two spectrally distinct colored formulations of CIS/ZnS QDs, greenish-QDs (G-QDs) with band edge emission at 530 nm and red-QDs (R-QDs) with band edge emission at 650 nm, were deposited sequentially as adjacent microdroplets on a glass substrate. Each QDs formulation displays a characteristic dual-emission profile, originating from the coexistence of a core-related band edge transition and a deeper shell-mediated state.^51–54^ In both formulations, QDs are capped with poly­(methyl methacrylate) (PMMA), which acts as a stabilizing matrix to support droplet integrity and longevity.^55^ These two emissions yield two spectrally distinct peaks for each QDs formulation, as shown in the individual PL emission spectra in the left (G-QDs) and right (R-QDs) panels of Figure 1b, at a 320 nm excitation wavelength. The sequential drop-casting approach ensures that the two droplets remain compositionally independent while making an edge-to-edge interface at the middle panel of Figure 1b, forming a well-defined multimodal emission profile. This configuration differs from physical mixing: instead of blending the QDs into a single emissive region, each droplet maintains its characteristic optical properties, resulting in two spatially separated yet optically addressable domains. For comparison, if the G-QDs and R-QDs formulations were physically mixed prior to or during deposition, both emitters would occupy the same spatial region, leading to increased spectral overlap and possible reabsorption interactions between nanocrystals. Such mixing can reduce peak distinguishability and limit the number of independent spectral features that can be extracted for encoding. In contrast, the side-by-side deposition strategy preserves spatial separation between the two emissive domains, allowing their emissions to be simultaneously detected while maintaining spectral independence. This configuration therefore provides improved peak resolution and a larger set of extractable optical features for fingerprint generation, as can be seen in Figure S1, Supporting Information (SI). The zoomed-in optical micrographs in Figure 1b highlight this spatial organization. The left region displays the characteristic greenish color of the G-QDs droplet, while the right region shows the deeper red color from the R-QDs droplet, presenting the random distribution of particles. This heterogeneous particle distribution originates from the stochastic dynamics of droplet spreading and solvent evaporation during the deposition process. Evaporation-driven capillary flows and local surface interactions lead to small but irreversible variations in the local nanocrystal density and arrangement across the deposited domains.^56,57^ As observed in the PL micrographs of Figure 1b, these variations produce spatially nonuniform emission patterns, which constitute the physical origin of randomness exploited for optical fingerprint generation. Since these are nanoparticles, it is impossible to achieve the same arrangement of particles when they are spread across any substrate. The contrast between the two regions arises from the intrinsic color difference of the G-QDs and R-QDs formulations, indicating that each droplet maintains its distinct optical characteristics after deposition. When the excitation spot is positioned to simultaneously illuminate both droplets at the interface, the detector records a combined emission profile, shown in the central spectrum of Figure 1b. This spectrum contains full emission contributions from both domains, effectively merging the dual peaks of G-QDs and R-QDs into a unified multicomponent response. Importantly, this composite emission does not arise from chemical interaction or alloying; rather, it results purely from the spatial dots arrangement, where both G/R-QDs domains contribute independently to the detected PL emission. This approach introduces a controlled method to access multiple spectral signatures from a single excitation wavelength, laying the foundation for the high-dimensional optical fingerprinting explored in later sections.

**Figure 1:**
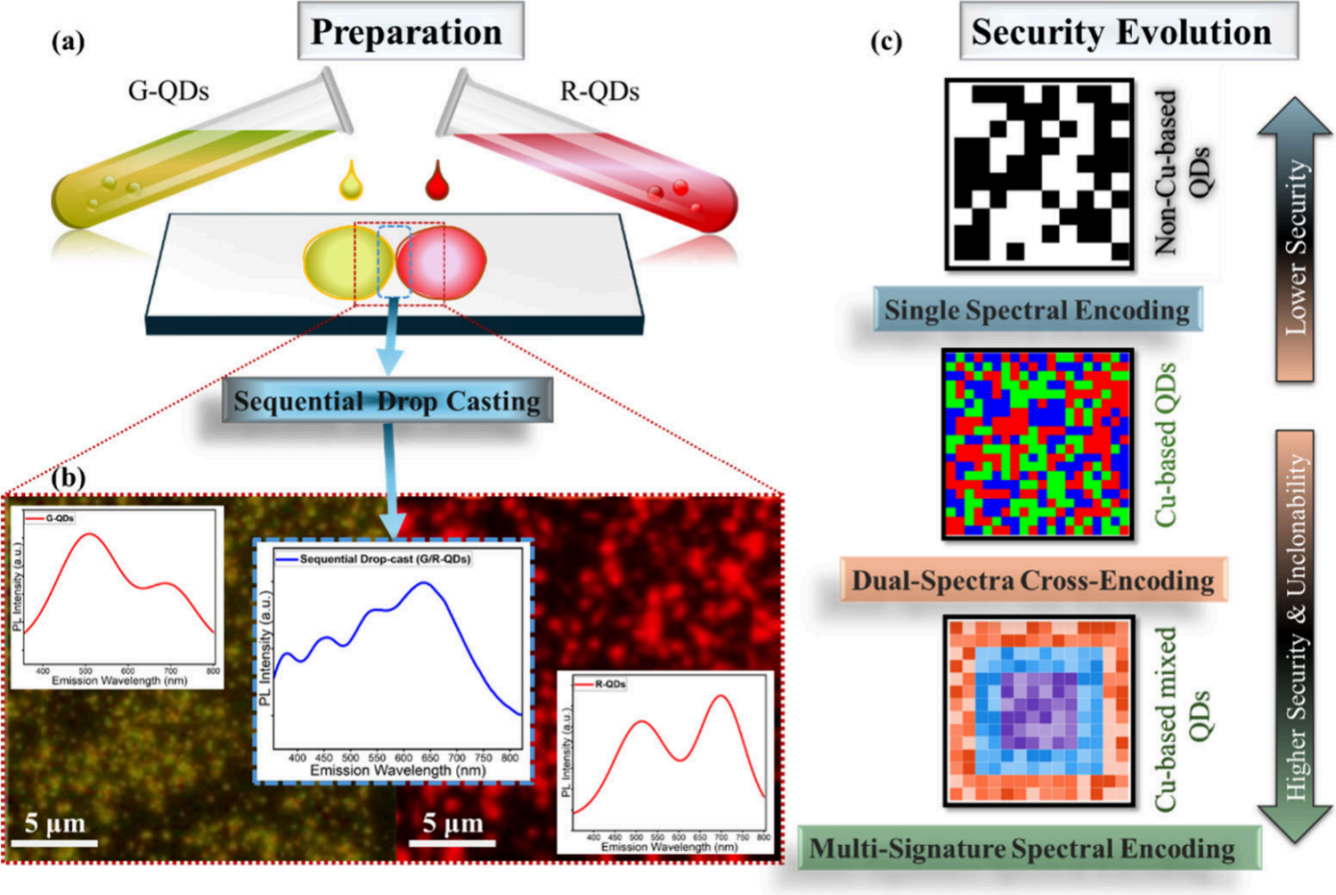
Sequential deposition of spectrally distinct colored formulations of CIS/ZnS. (a) Schematic illustration of the preparation process. (b) PL emission spectra from the individual G-QDs and R-QDs and the combined spectrum (G/R-QDs) obtained from the sequentially deposited region (along with the background of PL micrographs for G-QDs and R-QDs). (c) Conceptual illustration of security evolution in spectral PUF designs.

Figure 1c conceptually compares three levels of optical security that can be achieved with three different QDs-based tags: single spectral encoding, dual-spectra cross-encoding, and the multisignature spectral encoding implemented. In the single spectral encoding procedure, the tag is derived from the PL emission of cadmium-based QDs, cadmium selenide/zinc sulfide (CdSe/ZnS). The emission peaks for them at different excitation wavelengths are presented in Figure S2 (SI). The security metric is essentially determined by variations in the overall intensity of these emission maxima, which are very limited considering the features that could be extracted. As illustrated by the binary pattern in the top panel of Figure 1c, the tag is limited to grayscale levels derived from one emission only. This narrow spectral degree of freedom leads to low entropy, a reduced number of distinguishable states, and therefore lower security and higher clonability. In contrast, dual-peak emission, as observed in CIS/ZnS QDs, naturally provides more informative spectral content and hence a greater number of extracted features. These QDs exhibit two distinct emissions: a band edge transition associated with the core, and a deeper transition influenced by the shell and surface-related states; the emergence of these two peaks is excitation-dependent and can be seen in the plots of Figures S3 and S4 (SI), presenting the individual response of G-QDs and R-QDs, respectively.^51–53^ The coexistence of these emissive pathways produces a characteristic dual-band spectrum whose relative intensities, spectral positions, and excitation-dependent behavior offer multiple optical features for encoding. As shown in the middle panel of Figure 1c, this intrinsic dual-peak response delivers a richer spectral landscape than single-emission systems. Building on this foundation, the present work introduces a multipeak spectral emission, where two spectrally distinct colored formulations of CIS/ZnS are spatially arranged on a glass substrate. Each QDs formulation retains its own dual-peak behavior, and when their emissions are captured together at an excitation of 320 nm, the system presents four independent emission peaks originating from two spatially distinct G/R-QDs domains. The resulting optical output contains a broader set of peak-dependent features that can be computationally extracted and combined, enabling a higher degree of spectral diversity and fingerprint complexity. As represented in bottom panel of Figure 1c, this multipeak output forms the basis of the G/R-QDs multimodal emissions reported in this study, offering a platform for high encoding capacity optical authentication systems. This will be discussed in detail throughout this work.

### Excitation-Dependent Activation of G/R-QDs

A key advantage of this side-by-side deposition approach is that the optical response can be programmed by simply tuning the excitation wavelength without modifying the material or probing location. Figure 2 shows spectra collected from an edge-to-edge interface of the drop-cast distinct colored formulations of CIS/ZnS QDs while increasing the excitation wavelength from 250 to 345 nm with a 5 nm step. This experiment reveals four emissions, two from G-QDs and two from R-QDs, that are selectively activated depending on the excitation conditions. Plot 1 in Figure 2 shows the PL emission spectra using the shortest excitation wavelengths in the 250–270 nm range. Under these conditions, the emission originates exclusively from the band edge transitions of the two spectrally distinct colored formulations of CIS/ZnS QDs, G/R-QDs. As a result, only two well-defined emission peaks are visible: Peak A, centered around 650 nm, and Peak B, centered around 530 nm.^53,54,58^ These two peaks correspond directly to recombination within the respective CIS core of each QDs formulation. CIS/ZnS nanocrystals are well-known to exhibit a dominant band edge emission arising from electron-hole recombination within the CuInS_2_-rich core, which typically produces a single, composition-dependent emission band.^53,54^ This behavior has been consistently reported across multiple studies of CIS/ZnS core/shell QDs, where the emission wavelength is mainly governed by the core/shell stoichiometry and size.^54,58–62^ At the shortest excitation wavelengths, both G-QDs and R-QDs absorb strongly, and their band edge transitions dominate the carrier relaxation pathways. Because these transitions are the most efficient, they overshadow any weaker interface-associated paths, which remain below the detection threshold in this excitation window. Consequently, only the two intrinsic band edge peaks of the CIS/ZnS formulations are observed.^54,63,64^

**Figure 2:**
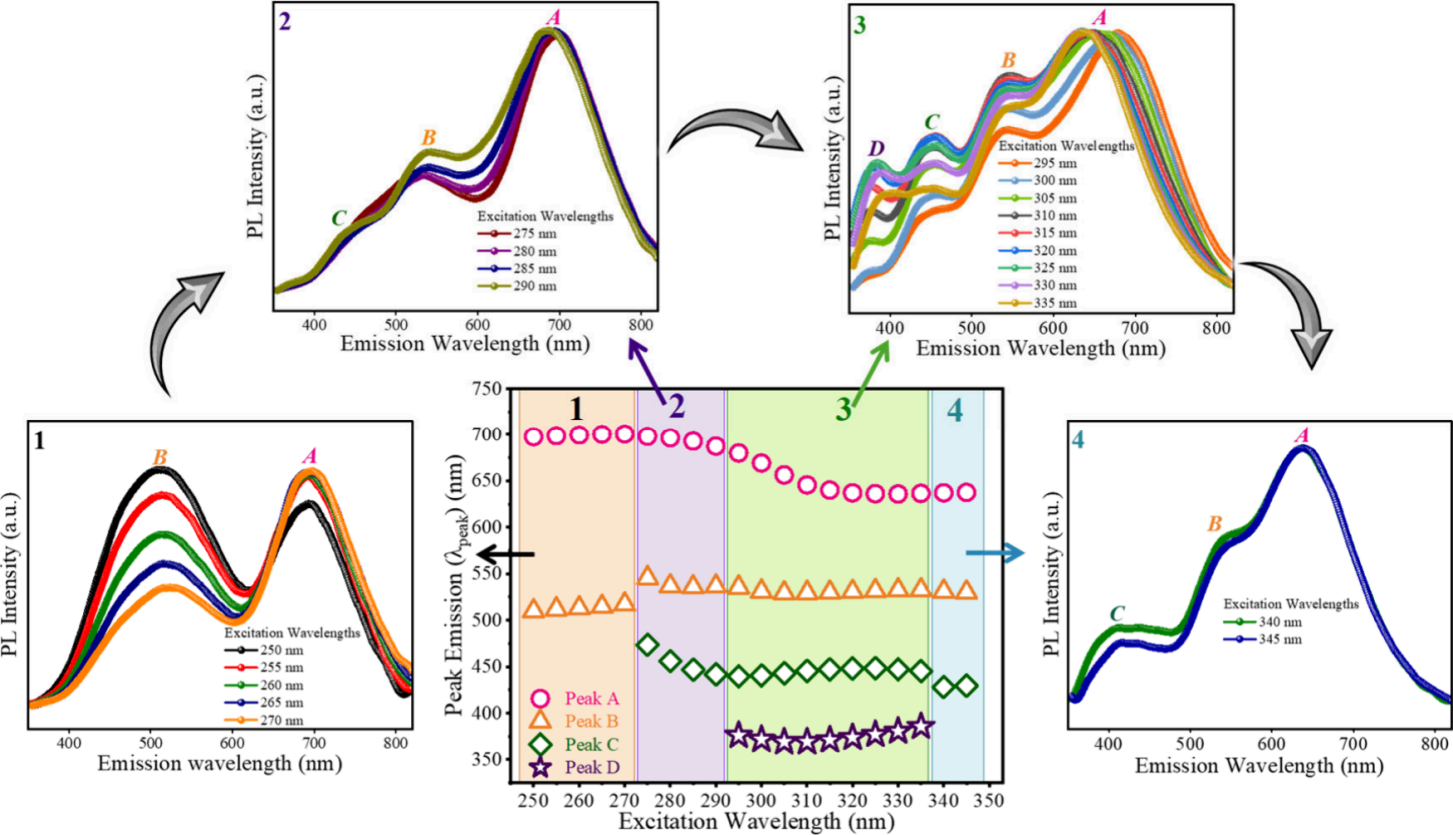
Excitation-dependent activation of multipeak emissions for the side-by-side deposition approach of G/R-QDs.

Following the excitation window where only the two band edge emissions of G-QDs and R-QDs are active, the next set of spectra reveals the appearance of an additional emission, Peak C at around 450 nm, which can be seen in plot 2 of Figure 2. This transition marks the point at which the excitation wavelength becomes sufficiently long (lower photon energy) to begin populating states beyond the core band edge levels of the two QDs formulations.^61^ A similar excitation wavelength-dependent reshaping of the PL emission spectrum, attributed to selective excitation of different substates, has been reported for CIS/ZnS nanocrystals by Hua et al.^65^ CIS/ZnS QDs possess multiple emissions associated with their core and graded core/shell interface.^51,66^ At shorter wavelengths, excitation occurs far above the band edge where both QDs formulations have strong absorption, leading to a dominant population of their respective core band edge states (Peaks A and B) after rapid relaxation. And, as the excitation wavelength increases, the absorption cross sections of the two CIS/ZnS formulations evolve differently. In particular, the R-QDs population exhibits stronger absorption in this intermediate range, enabling more efficient carrier generation in its core/shell interface-associated pathway, which gives rise to Peak C.^67^ Because the excitation energy is now closer to the absorption edge, the pumping becomes more selective, allowing this additional emission path to become visible without being overshadowed by the stronger band edge transitions. Thus, plot 2 in Figure 2 represents a selective activation window, where Peaks A and B still dominate but the interface-related emissions of one QDs formulation (Peak C) begin to contribute measurably.^68^ This marks the first step in the transition pathway from a simple two emissions to a more complex multipeak optical response.

As the excitation wavelength is further increased from the conditions corresponding to plot 2, the side-by-side QDs domains of G/R-QDs enter an excitation window where four distinct emissions (A–D) are simultaneously observed. In this range, the PL emission spectrum shows two higher-energy bands and two lower-energy bands associated with the G-QDs and R-QDs, indicating that both the core band edge as well as thecore/shell- and interface-related emissions of each formulation are now contributing measurably.^51,66^ This four-peak spectrum reflects a coherent combination of four emissions within the same material system: two band edge pathways, one from G-QDs and one from R-QDs, and two additional paths associated with their respective core/shell and interface regions or ZnS shell.^51,52^ Importantly, the sequential side-by-side deposition ensures that the two spectrally distinct colored formulations of CIS/ZnS remain separated yet are probed within the same excitation region. This prevents excessive spectral merging that can occur in fully mixed or alloyed systems and allows all four emissions to remain spectrally resolved in a single measurement. Optical and time-resolved studies on CIS/ZnS systems have similarly shown that maintaining a well-defined core/shell structure and controlled interface region leads to distinguishable contributions from the core and interface upon altering the excitation wavelength.^48,69–75^ From the perspective of encoding and fingerprinting, this four-peak excitation window is the most critical/suitable operating condition of this approach. Here, the spectrum is not only brighter but also richer in independent spectral features, as each of the four peaks provides its own intensity, position, and width and additional information is contained in their pairwise ratios and relatively dense spectral state of features. Plot 3 of Figure 2 therefore represents the point at which the sequential G/R surface design fully expresses its multisignature potential. By appropriately choosing the excitation wavelength, the system can be driven into a state where all of the distinct emissions are active and simultaneously accessible. It is this controllable four-peak emission regime that we subsequently utilize for quantitative peak fitting and for constructing the high-dimensional/entropy optical fingerprints presented in the next discussion.

For excitation wavelengths ≥340 nm, the spectrum collapses back to three peaks, as shown in plot 4 of Figure 2, and then toward the two dominant band edge peaks originating from the G-QDs and R-QDs. The PL emission profiles for the range beyond 345 nm can be seen in Figure S5 (SI). As reported for CIS/ZnS QDs, longer excitation wavelengths preferentially excite the lowest-energy absorption transitions, which funnel carriers predominantly into the band edge path.^64,65,76^ Under these conditions, the weaker core/shell- and interface-associated emissions that produced Peaks C and D are no longer efficiently populated. As a result, the spectra return to the simple dual-band profile defined by Peaks A and B (Figure S5a,b (SI)), mirroring the behavior observed in the first and second excitation windows.

The central plot in Figure 2 provides a consolidated view of how the four-peak emissions (λ_peak_) (A–D) evolve as the excitation wavelength is systematically increased from 250 to 345 nm. While the surrounding spectra illustrate the qualitative appearance of different emission signatures, this intensity-evolution plot quantitatively resolves the activation, coexistence, and suppression of each emission pathway. Such behavior is critical for understanding and later using the programmable spectral diversity required for optical PUFs. Other extracted parameters, such as the full width at half-maximum (FWHM) and peak intensity (*I*_peak_), are also plotted and presented in Figure S6a,b (SI), respectively. Unlike single-domain or chemically mixed QDs, where emission pathways often overlap or merge, our spatial separation of the G-QDs and R-QDs domains preserves the individuality of their core and core/shell emissions. As demonstrated in Figure 2, tuning the excitation wavelength allows these emissions to be selectively and jointly activated, enabling controlled transitions between dual-, tri-, and four-peaks emission states. This programmable activation of distinct pathways enables an expanded spectral basis from which high-dimensional optical fingerprints can be constructed.

### Spectral Features to Binary Fingerprint Generation

To translate the excitation tunable multipeak emission behavior shown in Figure 2 into a functional optical fingerprint, we focus specifically on the four-peak activation window where the G/R-QDs express their richest and most information-dense spectral state. This region is of central importance because it provides four independently addressable emissions whose combined behavior contains substantially more entropy and variability than the dual-peak regions. Figure 3 illustrates the full computational flowchart that converts these multipeak spectral responses into a structured, quantized optical fingerprint. Beginning with the extraction of clear peak-resolved parameters from the PL emission spectrum in Figure 3a, the workflow proceeds through features fusion and quantization in Figure 3b before generating a final geometric and binary representation of the unique optical identity of the QDs tag in Figure 3c. These stages of conversion transform the excitation-dependent optical response into reproducible and unclonable digital fingerprints, forming the foundation of our optical PUF system.

**Figure 3:**
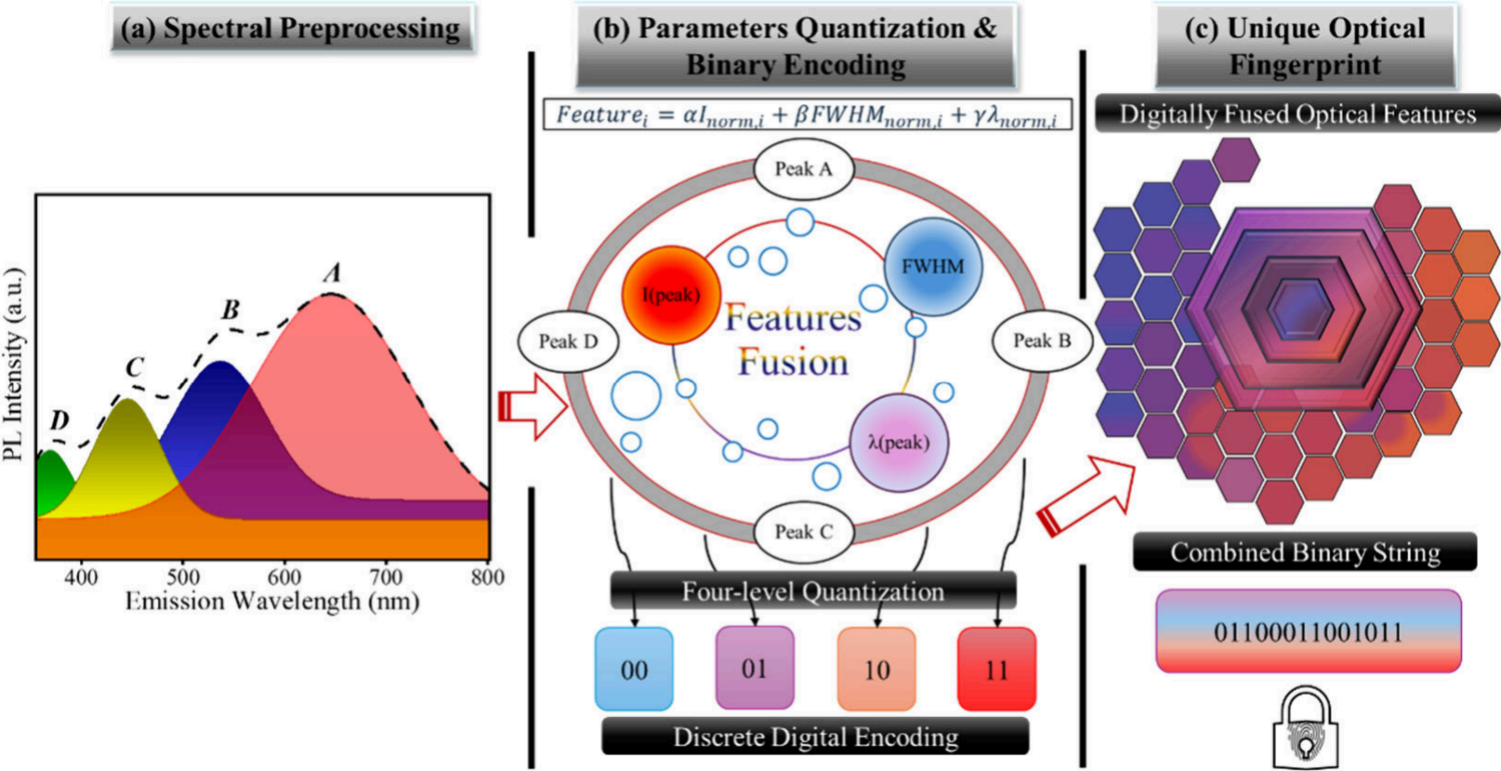
Workflow for extracting multiparameter optical features and generating an optical fingerprint from the four-peak G/R-QDs region. (a) Spectral preprocessing and peak isolation from the four-peak excitation range. (b) Computational features fusion, quantization, and binary encoding. Here, *i*: Peak A–D; α/β/γ: weighting factors for parameters. (c) Construction of the visual optical fingerprint from the binary bit string.

Figure 3a illustrates the first stage of the fingerprint generation workflow, where the raw PL emission data obtained from the four-peak excitation window, i.e., plot 3 in Figure 2, are prepared for features extraction. In this step, the recorded spectra undergo standard preprocessing operations including baseline correction, smoothing, and normalization. Each of the four emission peaks is then isolated and mathematically fitted using multipeak Gaussian functions to ensure the precise extraction of peak-specific parameters. This fitting step is essential for differentiating partially overlapping emission contributions and for ensuring that the parameters used in subsequent encoding stages reflect the intrinsic behavior of each emission rather than noise or measurement artifacts. The output of this preprocessing stage is a set of clean, peak-resolved spectral components, each associated with well-defined properties including *I*_peak_, λ_peak_, and FWHM. These extracted parameters form the backbone of the feature-vector representation used in the later quantization and binary encoding steps, ultimately enabling the generation of a unique, high-dimensional optical fingerprint.

Figure 3b represents the core computational stage, where the continuous spectral parameters extracted from the four peaks are transformed into discrete digital symbols suitable for fingerprint generation. After preprocessing, each of the four emission peaks is characterized by a set of parameters, which are normalized. Normalization here means that each parameter is scaled into a common range from 0 to 1 across all measurements. This step is crucial because it removes trivial scaling differences, i.e., absolute signal strength, instrument gain, or any slight wavelength offsets to ensure that intensity, width, and wavelength contribute on a comparable base. Without normalization, a parameter with larger numerical values would dominate the others in any arithmetic combination, even if physically it is not the most informative. These quantities capture features of the emission such as *I*_peak_, λ_peak_, and FWHM.

1 $${\mathrm{feature}}_{i}=\alpha I_{\mathrm{norm},i}+\beta {\mathrm{fwhm}}_{\mathrm{norm},i}+\gamma \lambda_{\mathrm{norm},i}$$

The fusion equation (eq 1) compresses these three normalized descriptors into a single scalar feature for each peak. In the equation, *i* denotes the index of the emission peak (*i* = 1–4 for Peaks A–D), and α, β, and γ are weighting coefficients that control the contribution of each parameter. For example, choosing a larger α gives more importance to fluctuations in peak intensity, whereas increasing β or γ makes the feature more sensitive to broadening or spectral shifts, respectively. In practice, these weights can be chosen empirically, by maximizing peak-to-peak separability or by setting up equal values when no prior preference exists. The key point is that any small change in any of the three underlying parameters will perturb feature_*i*_, making it a sensitive aggregate measure of the peak’s behavior. Mathematically, this linear combination can be viewed as projecting the three-dimensional parameter vector onto a single axis defined by the weight vector (α, β, γ). This has two important consequences for the optical PUF: First, it couples the parameters so that an attacker cannot treat intensity, width, and wavelength independently when attempting to clone the response. Second, it increases complexity because different combinations of small experimental variations in these three dimensions can map to distinct fused values. For the four peaks, this operation is performed for each peak, yielding a set of four fused features. When these are subsequently quantized into discrete bins, each fused feature contributes two bits to the final fingerprint. Thus, the equation is not a cosmetic mathematical step; it is the mechanism by which rich, multidimensional spectral information is distilled into compact but distinct numerical features that can be converted into a high-entropy binary code. The fused feature in eq 1 is used for digital encoding and entropy analysis; the hexagon in Figure 3c is generated directly from the three normalized descriptors (*I*_peak_, FWHM, and λ_peak_), not from the fused scalar. The continuous fused features are then passed through a four-level quantization scheme. The full dynamic range of each feature_*i*_ is partitioned into four non-overlapping intervals (bins). Each interval is associated with a discrete symbol, here represented as 00, 01, 10, and 11. Any given feature value is mapped to the corresponding bin and thus to a two-bit code word. Choosing four bins offers a deliberate balance between strength and complexity, as with only two bins (binary thresholding), each feature can assume just two states, making the system more tolerant to noise and limiting the number of distinct fingerprints. And with four bins, each fused feature can take one of four discrete levels, effectively encoding two bits per feature. For *N* fused features, this expands the combinative value from 2^*N*^ (simple thresholding) to 4^*N*^ possible symbol patterns, significantly increasing the potential number of unique fingerprints. These symbols form the fundamental units that will be concatenated and arranged in Figure 3c, which illustrates the final stage of the fingerprint generation process, where the quantized outputs are converted into a structured optical fingerprint using a three-layered hexagonal visual presentation. This captures the entire spectral identity of the G/R-QDs tag in a compact geometric form, allowing intuitive inspection while retaining the full information required for algorithmic verification. The hexagon is composed of three concentric layers, each corresponding to one class of spectral parameters extracted from the four-peak spectrum. The outer layer, middle layer, and inner layer encode the quantized *I*_peak_, the quantized FWHM values, and the quantized λ_peak_ from Peaks A–D, respectively.

Figure 4a expands upon the fingerprint generation concept by illustrating how the system responds to different excitation wavelengths within the four-peak spectral window, ranging from 235 to 275 nm. For each excitation condition, the quantized parameters from the four peaks are assembled into a 4 × *N* encoded grid, where each mini hexagon represents the quantized state of one peak at a specific excitation wavelength. This grid visually captures the multidimensional excitation–emission behavior of the G/R-QDs tag, embedding fine differences in intensity, FWHM, and wavelength derived parameters into a structured color-level representation. The central part shows how these grids are algorithmically fused into a single hexagonal fingerprint following the fusion rules established in Figure 3. This ensures that the final fingerprint retains all excitation-dependent spectral distinctions encoded across multiple conditions, resulting in a high-entropy and high encoding capacity fused representation. The encoding capacity of this tag was evaluated by considering the theoretical upper limit where each optical measurement contributes four excitation-dependent emission peaks, recorded under nine excitation wavelengths, with three spectral parameters extracted per peak.^77^ This yields a total of (4 peaks × 9 excitations × 3 parameters) = 108 independent spectral features.

**Figure 4:**
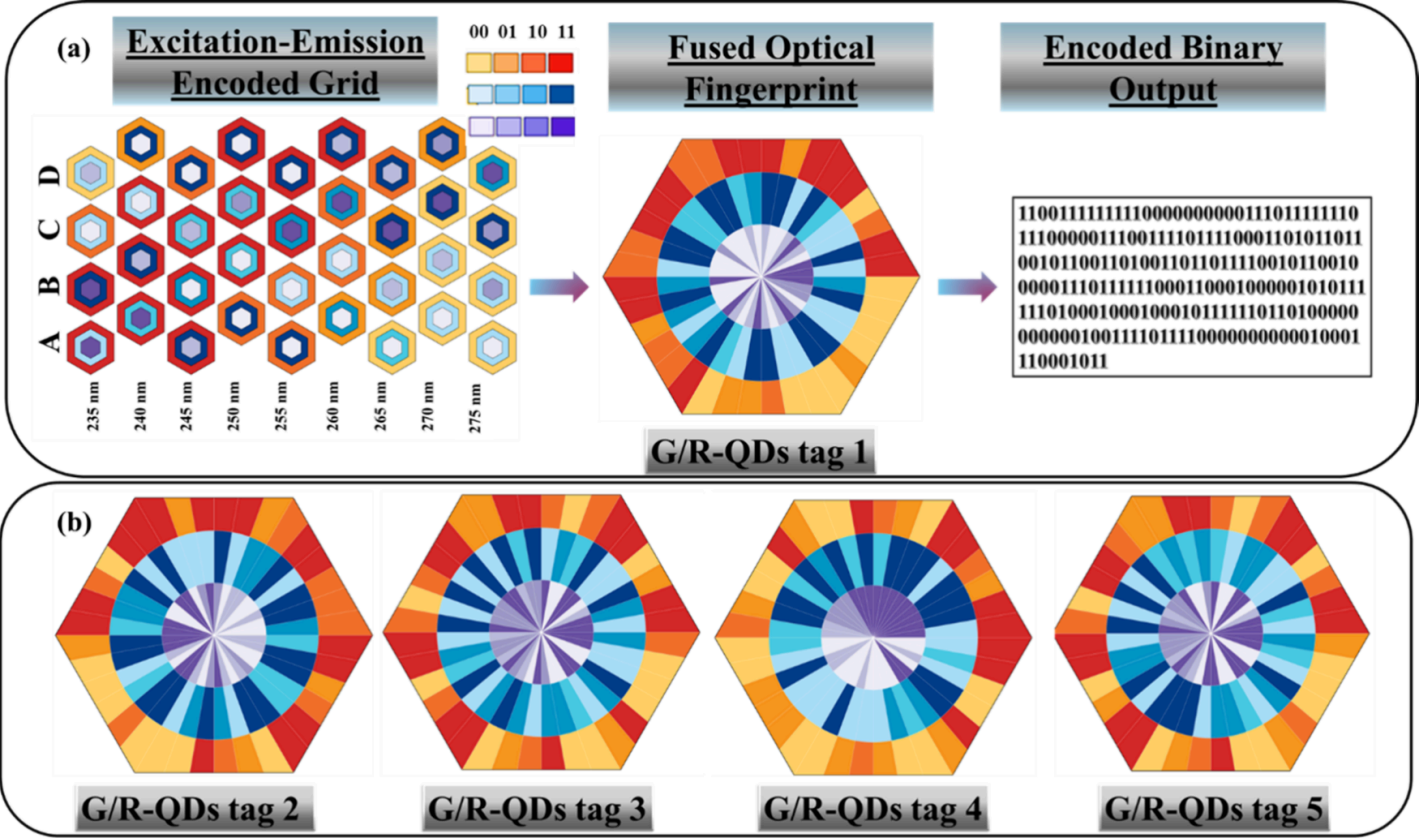
(a) Excitation–emission encoded grid to fused G/R-QDs tag generation with the encoded binary string and (b) final fingerprints of multiple G/R-QDs tags to verify the uniqueness of the system.

These features are binarized into a 216-bit fingerprint, corresponding to approximately two bits per feature. In the ideal case where every bit is perfectly random and independent and entropy = 1 per bit, the theoretical coding capacity (*C*_theoretical_) is

$$C_{\mathrm{theoretical}}=2^{216}\approx 1.05\times 10^{65}$$

This means that the system can generate on the order of 1 × 10^65^ distinct fingerprints, equivalent to a 216-bit key space.

For each layer of the hexagon, the circular space is divided into four angular segments, each representing one emission (A, B, C, D). The value assigned to each segment is determined by the quantized four-level features. Because each fused feature was mapped to one of four bins, each segment adopts one of four discrete shades, generating a visually distinct pattern unique to that tag. This presentation organizes the information systematically: the outer layer shows how bright each peak is relative to its dynamic range; the middle layer reflects the broadening or spectral sharpness of each peak; and the inner layer displays the behavior of the third parameter linked to the exact spectral position or its normalized shift. The progression from the outer to inner layer captures increasingly subtle spectral distinctions, producing a layered fingerprint that cannot be trivially mimicked by any single parameter manipulation. Once all 12 segments are assigned their respective quantization codes, the hexagon becomes the geometric embodiment of the fingerprint: a fixed arrangement of graded sectors whose appearance is governed entirely by the multidimensional spectral response of the G/R-QDs tag. This visual symbol is then accompanied by its corresponding binary string, formed by the two-bit codes of all fused features in a defined order (A → B → C → D for each layer). The resulting binary sequence functions as the digital authentication tag, while the hexagon provides a recognizable graphical identity for rapid visual comparison or machine-assisted recognition. The binary output on the right is obtained by concatenating the associated two-bit quantization symbols in a predefined order, yielding a stable and tag-specific digital fingerprint. Together, the three-layered hexagon and the binary string represent a parallel dual-format fingerprint optimized for digital comparison, error correction, and storage. This error correction is explained in the digitization part of the Experimental Section in detail. This dual encoding strengthens the unclonability of the optical PUF, ensuring that each tag yields a high-entropy, multilayered signature that is not possible to replicate without reproducing the exact spectral characteristics of the underlying QDs. And for this, Figure 4b evaluates how the fingerprint behaves across multiple identical tags. Each tag undergoes the same preprocessing, fusion, and quantization steps described in Figures 3 and 4a. Even though all tags are made from the same G/R-QDs materials and processed in the same way, each one still generates its own unique fingerprint. These differences originate from unavoidable nanoscale heterogeneities in droplet spread, domain boundaries, random distribution of nanoparticles, surface roughness, and interface microstructure, the features that are impossible to control or replicate intentionally.^32,55,78^ The resulting hexagonal patterns are therefore deterministic for each individual tag but uniquely different across the set of different tags, demonstrating the core requirement of an optical PUF, that is, the uniqueness, unpredictability, and resistance to cloning from tag to tag. This property is beneficial because it arises from the inherent disorder in QDs assemblies without requiring engineered randomness.

### Performance Evaluation of the G/R-QDs Tags

To quantitatively assess the performance of the proposed optical PUF, we evaluated the distinctiveness of the generated fingerprints. This section presents the core statistical metrics used to verify the uniqueness, repeatability, and randomness across all tags. Following the binarization step that converts the extracted spectral parameters into a compact digital representation enables the use of Hamming distance (HD)-based comparison, which is widely employed in PUF systems for evaluating uniqueness, reproducibility, and randomness. This transformation allows for rapid authentication while maintaining robustness against minor measurement fluctuations. Figure 5a presents the pairwise normalized HD matrix for the fingerprints generated from all G/R-QDs tags. As an example, for two tags *B*_*i*_ = (*b*_*i*__1_, ...,*b*_*iL*_) and *B*_*j*_ = (*b*_*j*__1_, ...,*b*_*jL*_) of length *L*, the normalized HD is defined as^12^

2 $${\mathrm{HD}}_{i,j}=\frac{1}{L}\overset{L}{\underset{k=1}{\sum }}\left(b_{ik}⊕b_{jk}\right)$$

where ⊕ denotes the XOR operation. This metric corresponds to the fraction of bit positions at which the two codes differ and therefore provides a direct measure of fingerprint dissimilarity on a [0, 1] scale. In the HD matrix, the diagonal elements are exactly zero (dark blue) as each tag is compared with itself. This confirms that the encoding and binarization processes do not introduce false variations when the same response is used in both entries of the comparison. The off-diagonal elements, which correspond to comparisons between different tags, are predominantly clustered around values of ∼0.5, as reflected by the light blue band away from the diagonal. An average intertag HD close to 0.5 is the expected behavior for uncorrelated binary strings and indicates that tags differ in approximately half of their bits. **^13,39^** Such behavior is characteristic of a high-entropy optical PUF, in which each G/R-QDs tag produces a statistically independent response and the probability of two tags sharing a similar code is vanishingly small. Importantly, the color distribution in Figure 5a does not reveal any obvious block structure or bands of systematically lower HD, which would indicate groups of G/R-QDs tags with correlated responses. Instead, the matrix appears homogeneous away from the diagonal, suggesting that tag to tag variability is dominated by intrinsic nanoscale differences in the distribution of G/R-QDs tags rather than by systematic fabrication artifacts. This uniformity across the matrix underlines the scalability of the proposed platform, allowing additional tags to be added without degrading the uniqueness of the overall fingerprints.

**Figure 5:**
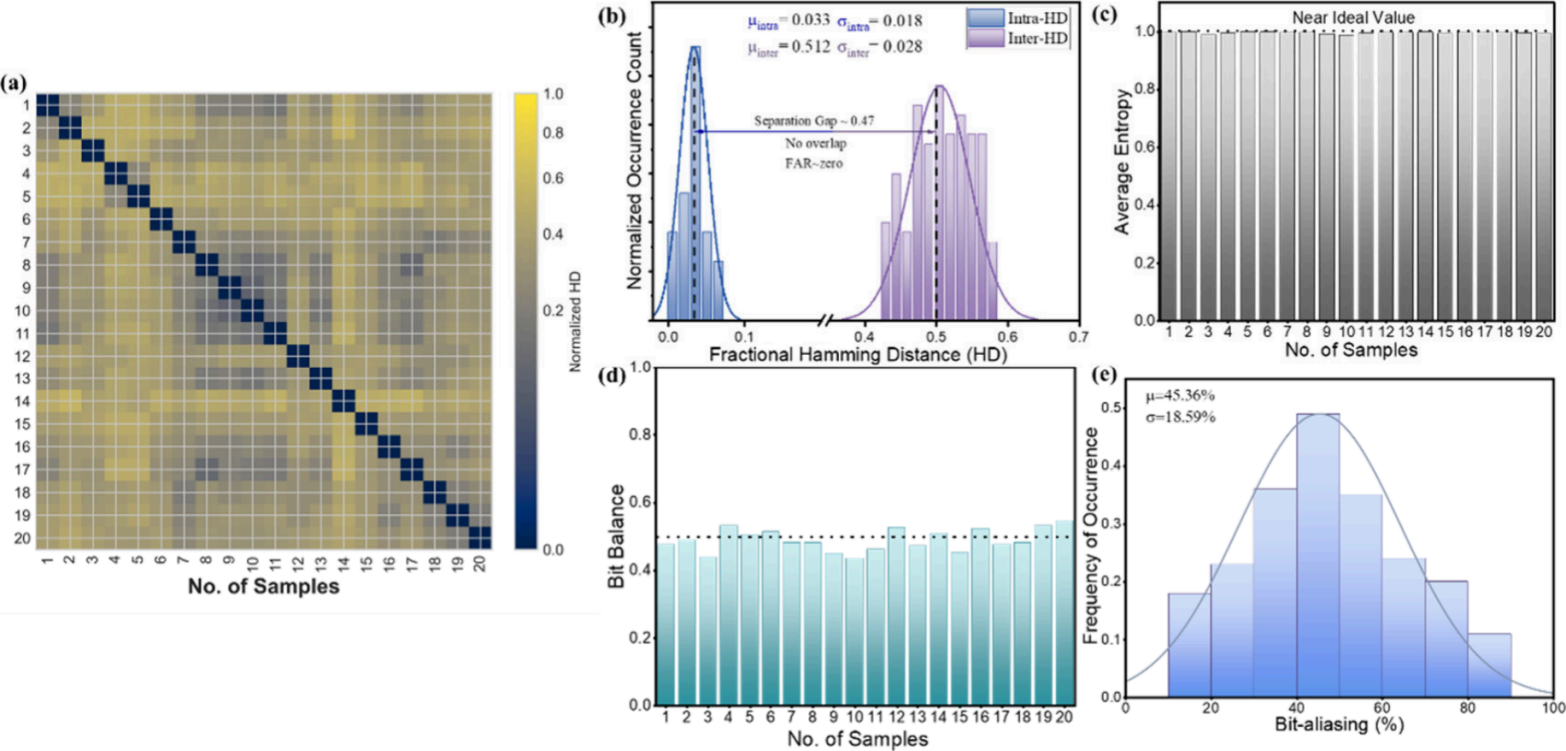
Quantitative evaluation of uniqueness, randomness, and bit-level statistics of the generated optical fingerprints. (a) Pairwise normalized Hamming distance (HD) matrix for all the G/R-QDs tags. (b) Distribution of inter- and intra-tag HD values extracted from (a). (c) Average Shannon entropy calculated at each bit position. (d) Bit balance across all bit indices and (e) bit aliasing distribution constructed from the bit-wise activation probabilities.

Figure 5b shows the corresponding inter- and intra-HD distributions, obtained by collecting all off-diagonal and diagonal HD values, respectively, from the matrix in Figure 5a. Each entry in this histogram represents the normalized HD between a unique pair of tags and therefore reflects the general statistical behavior of the G/R-QDs tags. The distribution exhibits a well-defined peak centered close to inter-HD ≈ 0.5, with a relatively narrow spread around this value. This behavior is consistent with the theoretical expectation for two independent binary strings of length *L*, for which the mean normalized HD is 0.5 and the variance decreases as 1/*L*.^12^ The close agreement between the empirical histogram and the curve overlaid in Figure 5b indicates that fingerprints generated from different G/R-QDs tags behave as statistically independent fingerprints. In practice, this means that the probability of predicting or modeling one fingerprint from another is not possible and that no tags exhibit any similarity with the rest of them.^13,39^ The sharpness of the peak further shows that uniqueness is not confined to a subset of tags but is uniformly manifested across the entire batch, supporting that the multipeak spectral features derived from the four-peak regime provide a rich, high-entropy source of randomness. The average inter-HD (*μ*_inter_) is 0.512 with a standard deviation (*σ*_inter_) of 0.028, which is almost identical with the ideal value of 0.5 for random and uncorrelated bit strings. This confirms the strong uniqueness and high pairwise separability among 20 different G/R-QDs tags. Since each tag has 216 bits and the bit HD (*D*) is not normalized, *D*_inter,mean_ = *μ*_inter_ × 216 ≈ 111 bits. So, on average, 111 bits differ between any two tags, out of 216, consistent with statistically independent binary codes. Further, to quantify the separation margin between these tags, we examined the distribution of all inter-HD. As we have 190 pairwise comparisons of 20 G/R-QDs tags, the lower and upper bounds of the intertag separation can be obtained: HD_min and max_ = *μ*_inter_ ± 2*σ*_inter_ = 0.456 and 0.568. In bit units of a 216-bit fingerprint, these correspond to *D*_min and max_ = HD_min and max_ × 216 ≈ 99 and 123 bits. Thus, the smallest observed intertag distance is approximately 0.456, which corresponds to nearly 99 differing bits. Even the two most similar tags therefore disagree in ∼46% of their bits. At the upper end, the largest intertag distance reaches 0.568, i.e., about 123 differing bits, confirming that pairs of fingerprints can diverge by more than half of their bit positions. Likewise, the intra-HD (*μ*_inter_) is 0.033 with a standard deviation (*σ*_intra_) of 0.018, with the broad separation window from almost 99 to 123 differing bits indicating that no two fingerprints in the data set approach similarity levels that could lead to accidental misclassification, guaranteeing an extremely low false-acceptance probability. The decision threshold adopted in our system is *T* = 0.20, which corresponds to the fact that two fingerprints are considered identical only if their normalized HD is ≤0.20. Since the smallest intertag distance is 0.456, all intertag comparisons lie well above the acceptance threshold. Consequently, the false-acceptance rate (FAR) is equal to zero, as no two different G/R-QDs tags were ever close enough to be misclassified as the same, as shown in Figure 5b, demonstrating that the uniqueness margin provided by the four-peak fingerprints is more than sufficient to guarantee zero false matches. The system therefore exhibits a strong uniqueness margin, consistent with the behavior expected from high-entropy, statistically independent binary codes.

Figure 5c presents the average Shannon entropy calculated at each bit position across all fingerprints generated from 20 different G/R-QDs tags. The entropy at a given bit index reflects how unpredictable that bit is when considering the entire population of the G/R-QDs tags. For a bit position with a probability *p*_*k*_ of being “1”, the Shannon entropy is defined as^12^

3 $$H_{k}=-p_{k}\log_{2}\left(p_{k}\right)-\left(1-p_{k}\right)\log_{2}\left(1-p_{k}\right)$$

An entropy value of 1 corresponds to a perfectly unpredictable position, which means the bit takes the values “0” and “1” with equal likelihood across tags, while values significantly below 1 indicate bias or redundancy. The plot in Figure 5c shows that the entropy values for nearly all of the bit positions lie close to the ideal limit. This behavior demonstrates that the quantization of multiparameter spectral features into binary form does not produce structurally biased bits. Instead, each bit position captures independent aspects of the underlying optical variability, resulting in a fingerprint in which information is broadly distributed rather than concentrated in a particular subset of positions. The high and uniform entropy profile across the fingerprint length confirms that the four-peak PL emission regime provides rich, high-dimensional features that are efficiently preserved during quantization. Importantly, none of the bit positions exhibit entropy collapse, indicating that no bit is stuck at a constant value. This balanced distribution of randomness across the entire bit string is essential for achieving a large effective encoding capacity and ensuring that the fingerprint cannot be easily reconstructed by an adversary. Experimentally, the effective encoding capacity is calculated by the measured entropy of the binary sequence. From the bit-wise entropy distribution in Figure 5c, the average entropy (*H*_avg_) per bit is ∼0.99, and for a 216-bit fingerprint, the number of effectively random bits is therefore

$$N_{\mathrm{eff}}=H_{\mathrm{avg}}\times 216\approx 213.84\;\mathrm{bits}$$

The corresponding experimental capacity *C*_exp_ will be

$$C_{\exp}=2^{N_{\mathrm{eff}}}=2^{213.84}\approx 2.36\times 10^{64}$$

Thus, the fingerprint behaves like a 214-bit cryptographic key, retaining approximately 99% of the theoretical 216-bit encoding capacity. These results highlight that the system not only expands the available features but also preserves near-ideal randomness after binarization, enabling a large encoding capacity suitable for secure authentication.

Figure 5d further examines the bit balance across all positions in the generated fingerprints. Bit balance measures the proportion of G/R-QDs tags in which a given bit position takes the value “1”. For an ideally unbiased fingerprint, this proportion should hover around 0.5, meaning both binary states occur with equal likelihood when considering the entire set of tags.^12,39^ The plot shows that the bit values across all positions slightly fluctuate within a narrow band centered close to 0.5, with no bit consistently favoring either state. This behavior indicates that the quantization of the fused spectral features does not introduce systematic bias toward “0” or “1” at any particular position. Instead, each bit reflects a balanced mixture of both outcomes across the tag. Such uniformity across the fingerprint length is essential for avoiding bias, which can otherwise reduce the effectiveness and make certain portions of the code more predictable. In the context of authentication and anticounterfeiting, balanced bits ensure that every section of the code contributes equitably to the uniqueness of the fingerprint, preventing scenarios where only a small subset of bits carries most of the distinguishing power. The near-flat distribution across all bit positions confirms that the encoding protocol preserves the statistical fairness of the binary representation and supports the generation of fingerprints that behave as unbiased, uniformly distributed binary sequences.

Finally, Figure 5e summarizes the bit aliasing behavior of the generated fingerprints by compiling the bit-wise activation probabilities across all positions into a single distribution. Bit aliasing quantifies how frequently the same binary value appears at a given position when comparing fingerprints from different tags. For each bit index *k*, the occurrence probability of a “1”, denoted as *p*_*k*_, is first computed across the entire set of G/R-QDs tags. These individual probabilities are then gathered into a single probability histogram, producing the distribution shown in Figure 5e. For a strong optical PUF, the bit aliasing distribution should be centered around 0.5, indicating that across the set of tags, no bit position persistently favors a particular state. A distribution that skews toward 0 or 1 would imply that some bits behave deterministically across tags, reducing the overall variability and diminishing the strength of the fingerprint. In contrast, the distribution observed here is tightly clustered around the center, with most entries lying close to *p*_*k*_ = 0.5. This indicates that the binary outcomes across the fingerprint are not dominated by fixed structural properties or fabrication trends but instead arise from genuinely G/R-QDs random distribution in the multisignature PL emissions. The shape of the bit aliasing distribution provides an overall view of randomness that complements the bit balance analysis. While bit balance evaluates each bit individually, bit aliasing shows the collective behavior of all bit positions and verifies that the encoding procedure does not produce any systematically weak fingerprint. A centered and symmetric distribution confirms that the fingerprint set exhibits healthy variability across tags, ensuring that no particular bit position is disproportionately influential or inherently more predictable.

In addition to the demonstrated performance, several characteristics of this optical PUF system should be considered for practical implementation. The optical response is spatially dependent, particularly at the G/R-QDs tag interface region, which requires controlled illumination conditions to ensure consistent readout. Additionally, the stochastic nature of the drop-casting process contributes to the intrinsic randomness of the tags but may introduce variability in the interface morphology that requires further optimization for uniform and large-scale production. The extraction of spectral features relies on peak fitting and computational encoding; maintaining algorithmic consistency across different measurement platforms remains an important consideration, although strong quantization, thresholding, and error correction strategies are implemented. These aspects represent system-level and implementation-oriented challenges, while the fundamental multispectral encoding mechanism demonstrated in this work remains unaffected.

## Conclusion

This work establishes a practical route for generating a complex optical QDs tag by using the multi-PL emissions from two spectrally distinct colored formulations of CIS/ZnS QDs arranged in a simple side-by-side configuration. The four-peak response produced under multiwavelength excitation provides a rich spectral landscape that can be reliably transformed into compact binary representations without sacrificing statistical integrity. Beyond demonstrating strong intertag separation and stable intratag reproduction, the analysis highlights that the underlying photophysical diversity of the Cu-based QDs can be systematically harnessed to construct multiple features suitable for secure authentication. The fingerprinting framework presented here offers two key advantages for real-world applications. First, it relies on solution-processable, environmentally benign emitters that are compatible with flexible substrates and scalable deposition techniques. Second, it maintains its strength under binarization, ensuring that the resulting codes remain resistant to replication and experimental noise. These attributes, combined with our experimentally supported analysis, position the multipeak QDs fingerprint as a compelling platform for lightweight security primitives.

The proposed multispectral system is particularly suitable for applications requiring high-security authentication, such as anticounterfeiting labels for high-value products, secure supply chain verification, and authentication of sensitive components. The use of cadmium-free QDs and solution-based fabrication methods also provides compatibility with scalable manufacturing processes, enabling potential integration into printed security labels, optical authentication devices, etc. This system offers key advantages including environmentally benign materials, scalable fabrication, and high-dimensional spectral encoding that produces statistically robust fingerprints. Future efforts will focus on accelerating readout, integrating smartphone-based acquisition, and extending the approach to multiplexed approaches to further enhance encoding capacity and maintain the near-ideal entropy. Overall, the strategy demonstrated here lays the groundwork for practical, high-entropy optical PUFs derived from simple nanomaterial designs.

## Experimental Section

### Materials and Chemicals

CIS/ZnS core/shell QDs were purchased from NNCrystal US Corporation. The formulation with band edge emission at 530 nm (G-QDs) possessed a 2.0 ± 0.5 nm CIS core, a 2.0 nm ZnS shell, and a total particle diameter of 4.0 ± 0.5 nm, with an emission peak centered at 530 ± 15 nm. The other one with band edge emission at 650 nm (R-QDs) featured a 3.5 ± 0.5 nm core, a 2.5 nm shell, and an overall diameter of 5.8 ± 0.5 nm, emitting at 650 ± 15 nm. These two QDs stock dispersions were diluted in anhydrous toluene to desired concentrations prior to film fabrication and optical measurements.

Poly­(methyl methacrylate) (PMMA, Mw ≈ 15 000 g/mol, from Sigma-Aldrich, catalogue 20036-50G) was used as received.

### G/R-QDs Tags Preparation

For fabrication, CIS QDs, G-QDs and R-QDs, were first dispersed in toluene to obtain 0.5 mg/mL stock solutions. PMMA was added as a stabilizing matrix to enhance the stability of the QDs. Glass substrates were sequentially rinsed with acetone and isopropanol followed by nitrogen drying to remove surface contaminants. The G-QDs solution was deposited onto the cleaned substrate via drop-casting and allowed to dry under ambient conditions for approximately 15 min. After complete drying, the R-QDs solution was dropped-cast immediately adjacent to the first region, ensuring that the two dried QDs domains formed at direct edge-to-edge interface. After complete solvent evaporation, these G/R-QDs were encapsulated by placing a second clean glass substrate on top to protect the film from mechanical and environmental degradation.

### Characterization

PL emission measurements were performed using an Edinburgh Instruments FLS1000 fluorescence spectrometer equipped with a 450 W xenon arc lamp as the excitation source. Excitation wavelengths were scanned from 240 to 450 nm, and emission wavelengths were collected by using a photon detector (PMT-900). The emission spectra were collected from 350 to 820 nm. All of the measurements were conducted under ambient conditions. PL emission acquisition was carried out specifically at the edge-to-edge interface between the G/R-QDs, where optical coupling and multimodal emission are the most pronounced. The prepared G/R-QDs tag was mounted horizontally in a fixed-position holder to maintain consistent alignment during sequential excitation scans. Data collection was performed using Fluoracle software, and all spectra were background-corrected and smoothed prior to further analysis.

### PL Emission Spectra Fitting for Data Extraction

All PL emission spectra were analyzed using OriginPro 2024b. Gaussian peak deconvolution was applied to every data set to extract the *I*_peak_, λ_peak_, and FWHM. Multipeak analysis was performed using Origin’s “Multiple Peak Fit” module, where each expected emission component was preidentified based on the raw spectra. Initial peak positions were estimated from local maxima, after which nonlinear least-squares optimization refined all fitting parameters simultaneously. A standard Gaussian profile (eq 4) was employed for all peaks, where *y*_0_ is the baseline offset, *A* is the peak amplitude corresponding to the integrated peak intensity, *x*_C_ is the emission peak center wavelength, representing the emission maximum, and *w* is the standard deviation-related width parameter that determines the spread of the peak.

4 $$y\left(x\right)=y_{0}+A\;\exp\left[-\frac{1}{2}{\left(\frac{x-x_{C}}{w}\right)}^{2}\right]$$

For a Gaussian function, the FWHM is analytically derived from eq 5.

5 $$\mathrm{fwhm}=2\sqrt{2\;\ln2}w$$

This expression was applied automatically within Origin to calculate the FWHM for each fitted peak. All fitting results, including *I*_peak_, λ_peak_, and FWHM, were exported directly into Excel spread sheets. The compiled data sets were subsequently imported into Python for quantitative fingerprint generation including the calculation of HD metrics to assess the reproducibility and uniqueness of each optical PUF.

It can be noted here that the raw PL intensity can be affected by environmental factors, such as minor mechanical vibrations, excitation fluctuations, or optical alignment variations. Here, however, we are capping our tags properly, and second, the encoding algorithm does not rely on absolute intensity values. Instead, normalized intensity values are used, which significantly reduce the influence of experimental fluctuations during repeated measurements. In addition, the encoding scheme is based on multiple spectral parameters, including peak wavelength, FWHM, and normalized intensity, extracted from the emission spectra under different excitation conditions. The use of this multiparameter feature set ensures that the fingerprint generation depends not on a single spectral parameter but rather on the combined spectral behavior of the system, which improves the strength of the generated fingerprints.

### Digitization of Optical Fingerprint

The four-peak spectral response obtained under multiwavelength excitation was converted into a 216-bit fingerprint using a streamlined Python-based workflow. The binarization process converts continuous spectral features into discrete digital fingerprints, enabling efficient comparison between tags using the HD metrics commonly employed in PUFs authentication systems. For each excitation, the wavelength, FWHM, and intensity of all four peaks were extracted and assembled into a 108-element feature vector. The spectral features were obtained through peak analysis of the PL spectra, allowing individual peak characteristics to be resolved before the encoding step. These features were mapped onto a 2D intensity matrix and discretized through a quantization step to suppress minor fluctuations and enforce encoding states. The threshold values used in the binarization process were derived from the statistical distribution of the extracted spectral features, allowing the continuous parameters to be consistently mapped into discrete states without manual tuning of the individual thresholds. This statistical quantization ensures that small variations caused by measurement noise or minor fluctuations do not lead to unstable binary outputs. As a result, the binarization procedure produces reproducible digital fingerprints while preserving the intrinsic spectral variability of the optical response. A threshold-based quantization and error correction scheme was implemented in the computational algorithm to ensure robustness of the generated binary fingerprints against minor spectral fluctuations. The resulting binary matrix forms a compact, noise-tolerant digital fingerprint that preserves the multidimensional structure of the four-peak emission while enabling direct statistical and security analysis.

## Supplementary Material



## Notes

Comparison of PL spectra for two different deposition techniques, excitation-dependent PL emission spectra of a non-copper-based CdSe/ZnS QDs tag, excitation-dependent PL emission spectra of the G-QDs tag, excitation-dependent PL emission spectra of the R-QDs tag, extended excitation-dependent PL emission spectra of the G/R-QDs tag, and extracted spectral parameters for the four emission peaks (A–D) originating from the G/R-QDs region (PDF)
