# Supplementary material for: Multispectral Quantum Dot Tags for Advanced Anticounterfeiting Applications
Source: ACS Appl Nano Mater. 2026 Apr 11;9(16):7182–94. doi: 10.1021/acsanm.6c00386 (PMC13122586; doi:10.1021/acsanm.6c00386)
Supplement: Supplementary file 1 [file an6c00386_si_001.pdf]

Supporting Information

Multi-Spectral Quantum Dot Tags for Advanced Anti-Counterfeiting Applications

Syeda Ramsha Ali, Yueyu Guo, Soumya Sarkar, Kees De Groot, Nema M. Abdelazim***

School of Electronics and Computer Science, University of Southampton, Southampton SO17 1BJ, United Kingdom.

* sra1r23@soton.ac.uk ** nema.abdelazim@soton.ac.uk

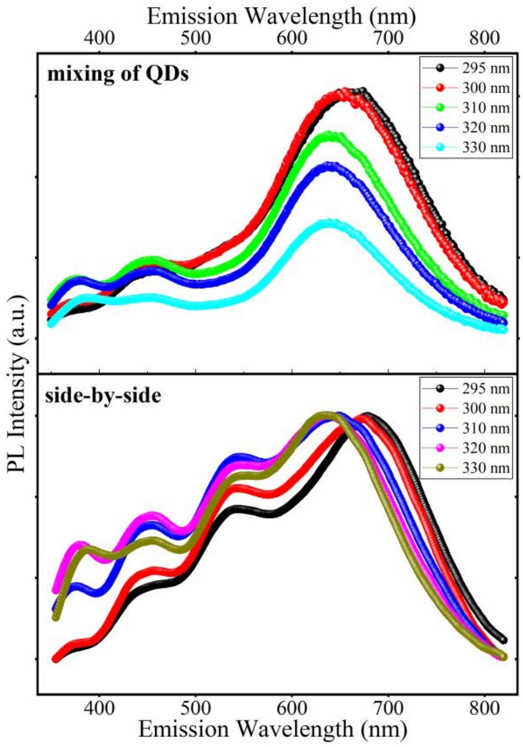

Figure S1: Comparison of photoluminescence (PL) emission spectra obtained from two configurations of CIS/ZnS QDs: *(top)* mixed G/R-QDs and *(bottom)* side-by-side deposited G/R-QDs

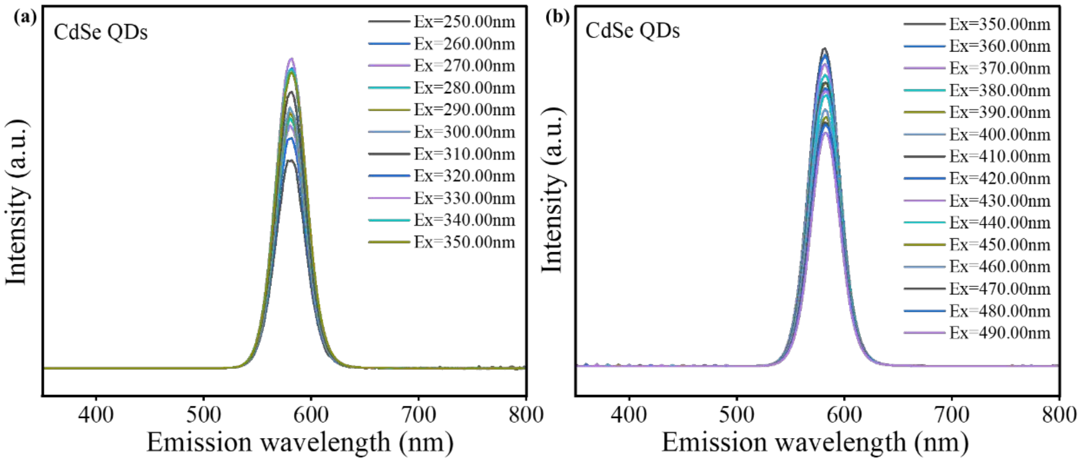

Figure S2: Excitation-dependent PL emission spectra of a non-copper-based, CdSe/ZnS QDs tag showing a single dominant emission peak across all excitation wavelengths.

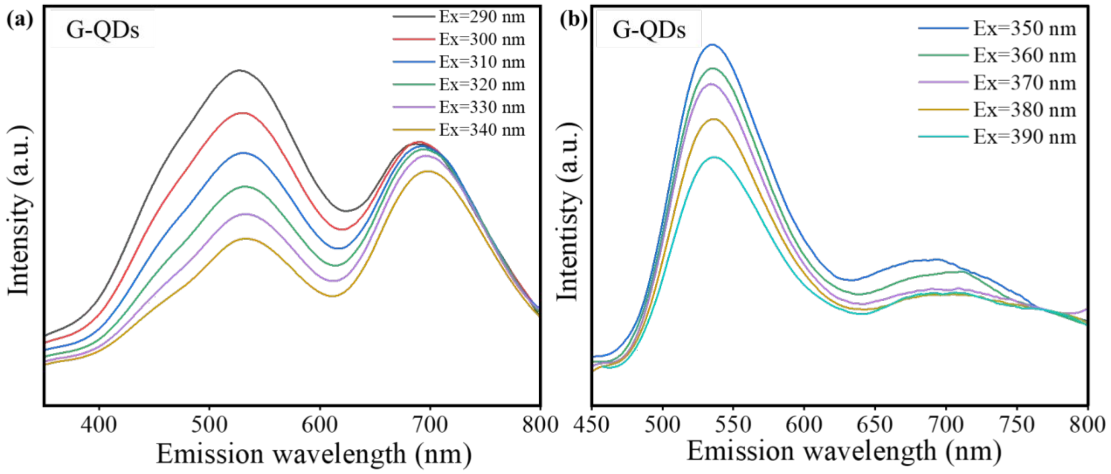

Figure S3: Excitation-dependent PL emission spectra of the G-QDs formulation, showing a clear dual-peak emission profile across all excitation wavelengths.

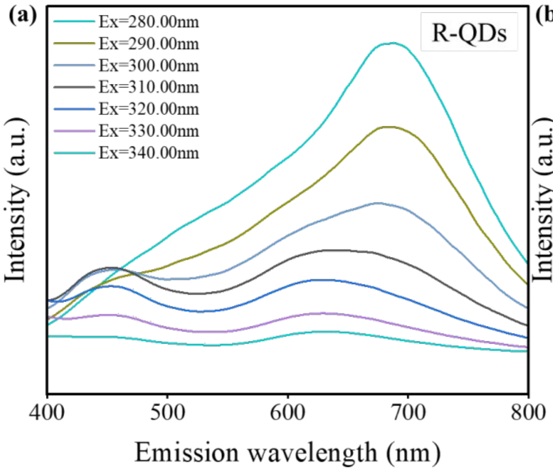

Figure S4: Excitation-dependent PL emission spectra of the R-QDs formulation. Two emission peaks are visible across all excitation wavelengths.

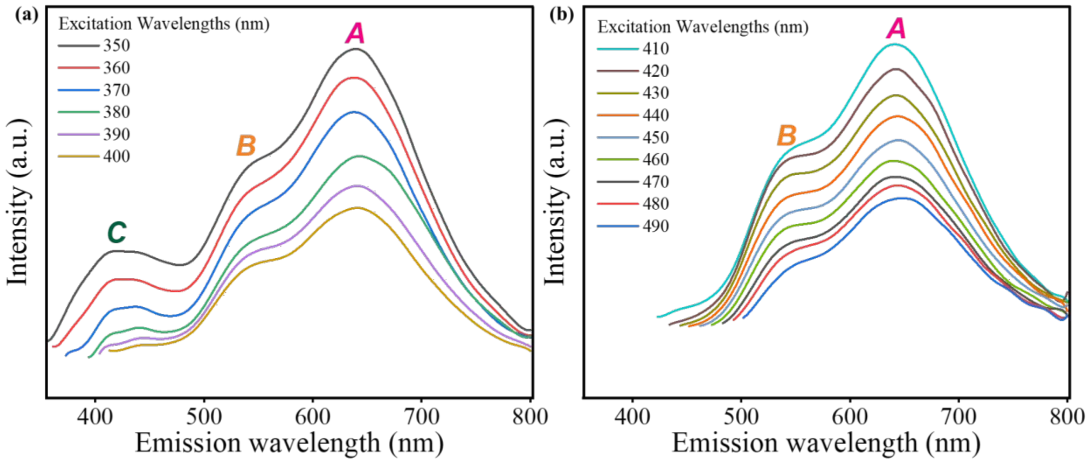

Figure S5: Extended excitation-dependent PL emission spectra of the side-by-side QD tag (G/R-QDs), highlighting the evolution of the four-peak composite emission to three-peaks and then to two-peaks, as the excitation wavelength increases.

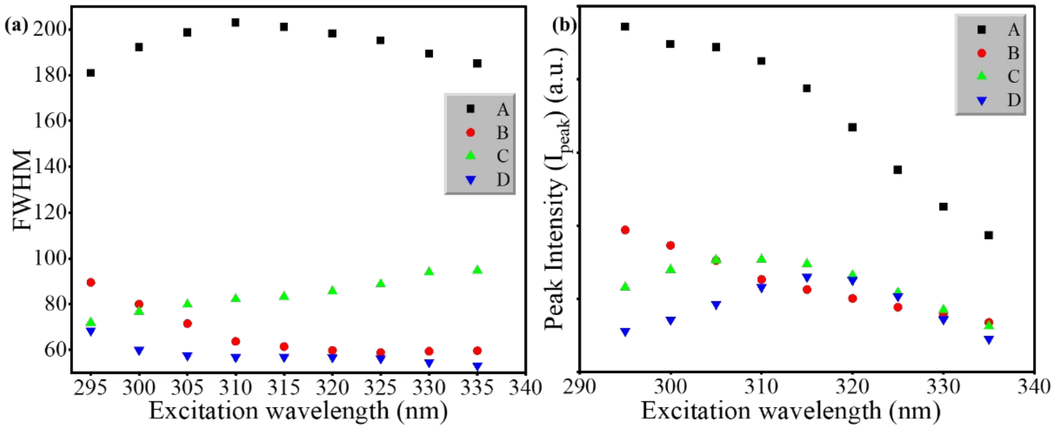

Figure S6: Extracted spectral parameters for the four emission peaks (A-D) originating from the G/R-QDs region. **(a)** FWHM values plotted as a function of excitation wavelength trends for each peak and **(b)** Corresponding I_{peak}, illustrating the excitation-dependent modulation of the four emission peaks.
